# Mid-Upper Arm Circumference Tapes and Measurement Discrepancies: Time to Standardize Product Specifications and Reporting

**DOI:** 10.9745/GHSP-D-21-00273

**Published:** 2021-12-31

**Authors:** Ritu Rana, Hatty Barthorp, Marie McGrath, Marko Kerac, Mark Myatt

**Affiliations:** a GOAL Global, Dublin, Ireland.; b Indian Institute of Public Health Gandhinagar, Gujarat, India.; c Emergency Nutrition Network, Kidlington, Oxford, United Kingdom.; d Department of Population Health, London School of Hygiene & Tropical Medicine, London, United Kingdom.; e Brixton Health, Llwyngwril, Gwynedd, Wales, United Kingdom.

## Abstract

In recent years, community-based management of acute malnutrition (CMAM) has revolutionized the care for children by increasing treatment coverage. Critical to the success of CMAM is early case identification. Mid-upper arm circumference (MUAC) measurement is a widely used, practical anthropometric measure used at the community level for the identification and admission of cases to appropriate treatment services. Globally, many organizations and government services use MUAC tapes for early case detection. However, there is no one universal MUAC tape specification, and it has been observed that using different MUAC tapes results in different measurements. In this article, we aim to: (1) present the measurement discrepancies; (2) discuss design specifications and their effect on case identification and admissions; (3) present a call to action to agree on common design specifications and standardized reporting. We hope this article will catalyze discussion and practical actions among nutrition and health stakeholders to ensure we have common MUAC tape design specifications so that all eligible at-risk children will get an equal chance to be identified early for critical treatment.

## Abstract (precis)

Mid-upper arm circumference (MUAC) is a widely used anthropometric measure to identify children with acute malnutrition. The use of different tapes of varied materials and thicknesses to measure MUAC has led to discrepancies. This indicates the need for global standardization of MUAC tape design.

## Key Messages

- There is no standard mid-upper arm circumference (MUAC) tape specification. Alerted by programmers in Ethiopia, we found using tapes of varied design results in measurement discrepancies and potential for systematic bias.
- This impacts case identification and the number of children eligible for treatment. At-risk children may be excluded from receiving the critical treatment they need.
- While some organizations use corrected tapes to account for design differences, many do not. Tapes used to validate current MUAC thresholds are not reported.
- We propose practitioners report on tape specifications used and call for global standardization of MUAC tape design and examination of possible implications for current MUAC thresholds.

## BACKGROUND

Globally, an estimated 47 million children under age 5 years are wasted and are at increased risk of mortality, morbidity, poor development, and long-term adverse effects (noncommunicable diseases).^1^^–^^3^ In recent years, community-based management of acute malnutrition (CMAM) has revolutionized the care for children (aged 6–59 months) by increasing treatment program coverage.^4^ Critical to the success of CMAM is early and effective case identification.^5^ Combining speed, low cost, ease of use, portability, and strong prognostic performance in identifying children at high risk of mortality/morbidity, mid-upper arm circumference (MUAC) measurement is a common anthropometric measure and is especially useful at the community level to identify and admit cases to appropriate health and nutrition services.^6^^,^^7^ MUAC is also recommended as a “reduced physical contact” approach in the context of coronavirus disease (COVID-19).^8^ In general, children with a MUAC of less than 115 mm are identified as severely wasted; those with MUAC between 115 mm and 125 mm are moderately wasted.

Globally, many ministries of health, international and national nongovernmental organizations are using MUAC tapes for early case detection in the community. However, there is not one MUAC tape specification, and it has been observed that using different MUAC tapes results in different measurements. In this article, we aim to: (1) present the measurement discrepancies; (2) discuss design specifications and their effect on case identification and admissions; (3) present a call to action to agree on common design specifications and standardized reporting.

## DIFFERENT TYPES OF MUAC TAPES AND SYSTEMATIC BIAS

**FIGURE 1:**
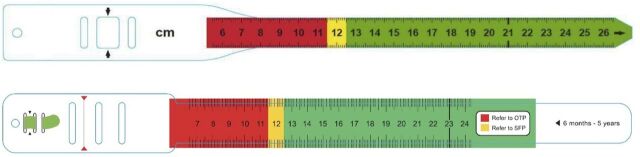
Two Mid-Upper Arm Circumference Tapes. Top: Type A (thickness: 180 microns; width: 17 mm; measurement: the scale measures the outside circumference of the tape); Bottom: Type B (thickness: 280 microns; width: 25 mm; measurement: the scale measures the inside circumference of the tape)

GOAL, an international humanitarian organization working in 13 countries across Africa, Latin America, and the Middle East, with headquarters in Ireland, is supporting a community-based program that uses MUAC to screen and admit children for wasting treatment in the Gambella refugee camps in Ethiopia.^9^ In Gambella, there are multiple screening opportunities (quarterly mass screening led by other organizations and monthly program screening by GOAL). Two types of MUAC (insertion) tapes are being used: type A and type B (Figure 1). A chance observation led us to note that health workers are sometimes faced with the problem of a child recording a MUAC measurement of 126 mm with one tape and the other measures 124 mm. Such cases create a dilemma: should a child be admitted to treatment services or not?

A chance observation led us to note that health workers are sometimes faced with the problem of a child recording a different MUAC measurement with different tapes.

**FIGURE 2:**
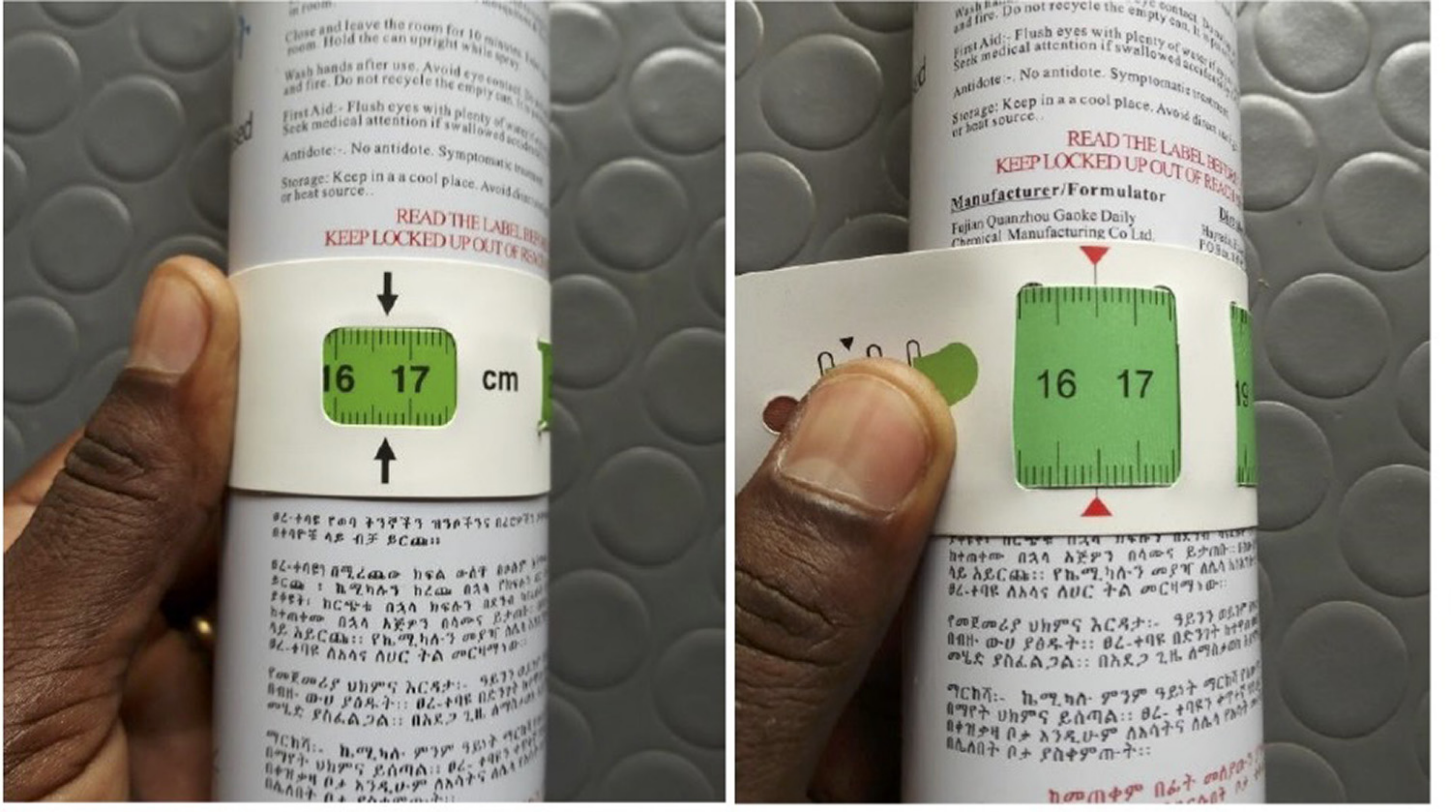
Measurements by Same Individual Using Same Object but Two Different Mid-Upper Arm Circumference Tapes. Left: Type A, 167 mm; Right: Type B, 165 mm

To confirm this measurement discrepancy, researchers at GOAL measured a solid cylindrical object using both type A and type B MUAC tapes. The findings showed a difference of 2 mm (as observed in the community), with type A giving measurement of 167 mm and type B of 165 mm (Figure 2).

Teams at GOAL suspected this discrepancy in measurement was due to the thickness of some tapes not being corrected for during the design process. This prompted a closer examination, which we elaborate on here.

The MUAC measurement is taken around the upper arm and thus, tapes made of thicker material add to the circumference. This leads to systematic bias/differences in MUAC measurements taken using different tapes (Table), unless this is corrected for in tape design—which currently is not the case.

**TABLE.** Systematic Bias (Built-in Error) in MUAC Tapes With Varying Thickness

Type A Tape (with material thickness of 180 microns)	Type B Tape (with material thickness of 280 microns)
Example 1: On a 100 mm true circumference, we have: D = C/π = 100/3.14159 = 31.8310	
C = π × (31.8310 + 2^a^ × 0.18) = 101.131 mm Built-in error of about 1.13 mm	C = π × (31.8310 + 2^a^ × 0.28) = 101.759 mm Built-in error of about 1.76 mm
Example 2: If we consider a 200 mm circumference, we have: D = C/π = 200/3.14159 = 63.6620	
C = π × (63.6620 + 2^a^ × 0.18) = 201.131 mm Built-in error also 1.13 mm	C = π × (63.6620 + 2^a^ × 0.28) = 201.759 mm Built-in error also 1.76 mm

Abbreviations: D, diameter; C, circumference; π (pi); MUAC, mid-upper arm circumference.

a Need to add twice the thickness of the material (For more detailed explanation of why the error is constant despite different circumferences, see the “String Girdling Earth” mathematical puzzle).

This systematic bias has implications for case identification and admissions to appropriate treatment services. We present the following hypothesized example to simulate possible consequences.

Applying an error-free tape to a population of 10,000 children with a mean MUAC of 142 mm with a standard deviation of 14.5 mm, we would expect (using the PROBIT approach to estimating prevalence)^10^ to identify:

PROBIT (115, 142, 14.5) × 10000 = 313 cases

If we do this using a tape with a 2 mm error, we expect to identify:

PROBIT (115 - 2, 142, 14.5) × 10000 = 228 cases

This means that the 2 mm error excludes 313 − 228 = 85 children (or 85/313 = 27% of “true” cases) with true MUAC less than 115 mm. This example shows that a 2 mm error, which introduced underestimation of malnutrition, excluded a considerable proportion of children who are at elevated risk of mortality but would be likely to respond rapidly (and with reduced cost) to treatment from which they are excluded due to MUAC tape error. These issues indicate the need for global standardization of MUAC tape design. Toward such common global design specifications and standardized reporting, we propose the following recommendations.

1. There should be a fixed thickness of MUAC tape material with this accounted for in the ruler.
2. If organizations choose to use different tape thicknesses than the common recommendation, they should shift the ruler to account for this, ensuring measurement of the true MUAC.
3. Before use, MUAC tapes should be checked against known circumference solid cylinders, preferably ranging from 110 mm–130 mm. This calibration check should be a standard practice as per other medical-grade anthropometric tools.
4. All future work should document which tapes were used, as already reported with weight and length/height measurement scales in research.
5. Finally, since it is not reported which tapes many original MUAC validation studies used, we also note a need for broader examination of implications for current MUAC thresholds.

### Limitations

We acknowledge the following limitations. The technical aspects presented in this note are results of observations reported by GOAL staff who spotted the measurement discrepancies in the field. With the observations we had, we could only explore thickness and the position of the measurement scale/ruler. Effects of other design aspects, such as large/small tab, number of “buckles,” tape width, and measuring points/arrows, were not explored. However, some information on these design aspects is presented elsewhere.^7^

A few organizations (Médecins Sans Frontieres, Action Against Hunger, and GOAL) have developed, tested, and used corrected MUAC tapes that adjust the position of the ruler to account for material thickness and measure the inside circumference of the tape (which is the circumference of the arm being measured). The adjustment is easily calculated using circle geometry (circumference of circle = Pi (π) × diameter).

Since 2019, GOAL has been using a corrected MUAC tape design, with the ruler adjusted to account for material thickness. However, at the time of writing, MUAC tapes produced and distributed by several organizations remain uncorrected.

## CONCLUSION

We hope this article will catalyze discussions and practical actions among nutrition and health stakeholders with leadership by the relevant United Nations agencies to ensure all eligible at-risk children will get an equal chance to be admitted to timely, appropriate treatment.

## Notes

**Peer Reviewed**

**First published online:** November 12, 2021.
